# Inputs to prefrontal cortex support visual recognition in the aging brain

**DOI:** 10.1038/srep31943

**Published:** 2016-08-23

**Authors:** Jessica R. Gilbert, Rosalyn J. Moran

**Affiliations:** 1 Virginia Tech Carilion Research Institute, Roanoke, VA 24016, USA; 2 Bradley Department of Electrical and Computer Engineering, Blacksburg, VA, 24061, USA

## Abstract

Predictive coding models of brain function propose that top-down cortical signals promote efficient neural codes by carrying predictions of upcoming sensory events. We hypothesized that older brains would employ these codes more prominently given their longer repertoire of sensory experience. We measured the connectivity underlying stimulus-evoked responses in cortical visual networks using electroencephalography and dynamic causal modeling and found that in young adults with reported normal or corrected-to-normal vision, signals propagated from early visual regions and reverberated along reciprocal connections to temporal, parietal and frontal cortices, while in contrast, the network was driven by both early visual and prefrontal inputs in older adults with reported normal or corrected-to-normal vision. Previously thought of as exceptions to the rule of bottom-up signal propagation, our results demonstrate a prominent role for prefrontal inputs in driving vision in aged brains in line with lifespan-dependent predictive neural codes.

In Bayesian theories of neural function, our brain’s internal generative model of the world is constantly updated through alterations in neural connections and driven by consistent, repeated inputs from the sensorium^1^,^2^,^3^. According to predictive coding accounts of these model updates, learned environmental statistics are encoded by top-down connections as ‘priors’ and serve to constrain bottom-up signal propagation which carry prediction errors^4^,^5^. While alterations in neuronal activations and connectivity consistent with these effects have been observed during single-session perceptual learning tasks^1^,^6^, human aging represents a natural ecological extension where predictive coding theories remain to be tested. The predominant view of cortical visual processing posits that perceptions are formed following bottom-up hierarchical signaling of successively complex features of a visual scene. This view has been challenged by recent evidence of a dual-route network in object recognition^7^,^8^ where presumed magnocellular projections to frontal cortex have been shown to exert early top-down effects on signals from bottom-up visual pathways^8^. Several recent studies have highlighted the importance of top-down signaling from higher to lower levels of the visual system to enhance task-relevant information^9^,^10^, and the idea of top-down influences in visual perception can be traced to the Gestalt school of psychology. This top-down functional pathway may arise anatomically from the mediodorsal thalamic nucleus, which has strong reciprocal connectivity with the frontal cortex and has recently been shown to receive direct inputs from the retina^11^. This prefrontal route carries visual information at low spatial frequencies, offering a ‘gist’ of the visual scene to be filled-in with representational details carried through bottom-up hierarchical processing. This route may accommodate predictive deployments to the visual network and participate in our hypothesized age-related reorganization of perceptual circuitry.

Repetition priming, which varies the familiarity of a stimulus, is a powerful paradigm to explore changes in generative predictions^12^ that accompany aging. Behavioral and neural changes associated with priming are both temporally long lasting^13^ and found across the adult lifespan^14^. A network of brain regions has been identified in priming studies, with regions thought crucial to facilitated naming showing repetition suppression^13^,^15^. These include regions in the ventral stream, both anatomically early regions and more anterior regions of ventral temporal cortex, in addition to frontal regions^13^. Importantly, transcranial magnetic stimulation applied to left frontal cortex has been found to disrupt behavioral and neural repetition suppression^15^, providing converging evidence that frontal predictions interact with signals propagating feedforward through the ventral stream. In addition to the ventral stream, activation in the angular gyrus has been identified in a host of object naming studies^16^, with converging evidence from patients with damage to the angular gyrus (BA 39 and 40) showing impaired picture naming^17^.

Here, we used dynamic causal modeling (DCM) for electrophysiology^18^ to explore changes in event-related feedforward and backward connections important for object naming during a repetition-priming paradigm^19^. DCM provides a biophysical model of currents produced by interacting brain regions that are then matched to measured signals. The canonical microcircuit (CMC) model, which we use here, includes four distinct cell layer contributions to measured signals, allowing for more precise estimates of feedforward and backward connections^5^ and how these change as a function of age. The CMC includes excitatory and inhibitory connection parameters from superficial pyramidal cells, spiny stellate cells, deep pyramidal cells, and inhibitory interneurons. The model was developed to test predictive codes^5^, where bottom-up connections from superficial pyramidal cells could encode and carry prediction errors to spiny stellate cells and deep pyramidal cells in a feedforward manner up the cortical hierarchy, while deep pyramidal cells could carry top-down predictions in a feedback manner to both superficial pyramidal cells and inhibitory interneurons at hierarchically lower levels. This architecture was designed for modeling predictive coding within cortical hierarchies, as estimates of forward and backward signaling are provided. We predicted that advanced age was accompanied by a greater reliance on top-down predictions that serve to explain away feedforward prediction error signals propagating from early sensory cortices^12^.

## Results

**Figure 1:**
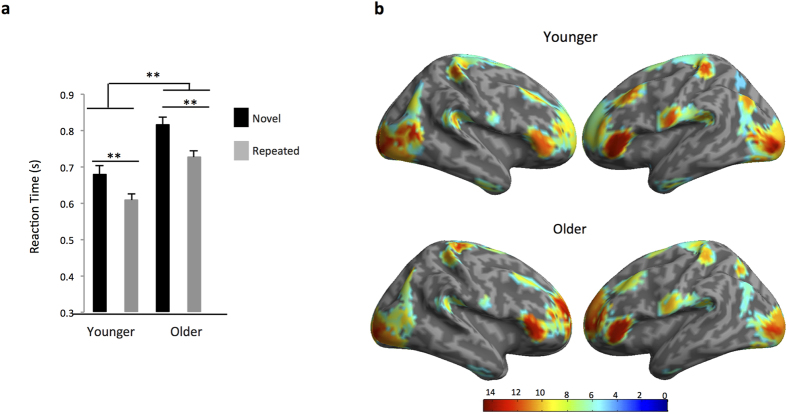
Behavior and Source-Localized Findings. (**a**) Behaviorally, both younger and older participants showed facilitated naming speed for repeated (Younger Mean: 608.8 msec +/− 0.02 SE; Older Mean: 727.0 msec +/− 0.02 SE) compared with novel (Younger Mean: 679.1 msec +/− 0.02 SE; Older Mean: 815.9 msec +/− 0.02 SE) pictures, with younger participants exhibiting significantly faster naming speed than older participants (t-value novel: −4.4 (42), p<0.01; t-value repeated −4.8 (42), p < 0.01). Importantly, we found no differences in the amount of priming (measured by Novel RT – Repeated RT) shown by younger (Mean: 70.2 msec +/− 11.3 SE) and older (Mean: 88.9 msec +/− 8.4 SE) participants (t-value: −1.3, p = 0.20). B. Wide-band (1–42 Hz) source-localized data for younger (top) and older (bottom) participants from 1–450 msec. One-sample t-tests were used to localize sources showing significant (p < 0.05 FWE corrected) positive increases in power.

We used a repetition-priming paradigm in tandem with DCM to measure changes in frontal cortex predictions, instantiated by feedback connections, as a function of aging. High-density electroencephalographic (EEG) recordings were acquired during the task from 23 younger and 21 older healthy participants. Behaviorally, both younger and older participants demonstrated facilitated naming for repeated compared with novel pictures, with younger participants exhibiting significantly faster naming speed than older participants (Fig. 1a). Importantly, we found no differences in the amount of priming (measured by Novel RT – Repeated RT) demonstrated by younger and older participants. We also measured explicit recognition memory performance separately by presenting participants with 50 novel and 50 repeated items and asking them to make old/new judgments. Behaviorally, both younger (recognition accuracy: 80.5 +/− 2.7) and older (recognition accuracy: 74.6 +/− 2.1) participants showed greater than chance recognition of previously seen items, with no significant differences between groups (students two-tailed *t* test p = 0.09), though there was a trend to significance. These behavioral findings, of recognition performance better than chance, suggest that older and younger subjects both saw and encoded the picture items as required for the priming analysis.

To infer the active sources or generators of the EEG, we used a multiple sparse priors routine and identified significant group-level activations in both the younger and older cohort consistent with previous reports on priming^13^,^14^,^15^ (Fig. 1b). The network included early visual cortex, anterior ventral temporal cortex, angular gyrus, and inferior frontal cortex. These four regions bilaterally were the focus of subsequent DCM analyses, due to their principle role in object naming and repetition priming.

**Figure 2:**
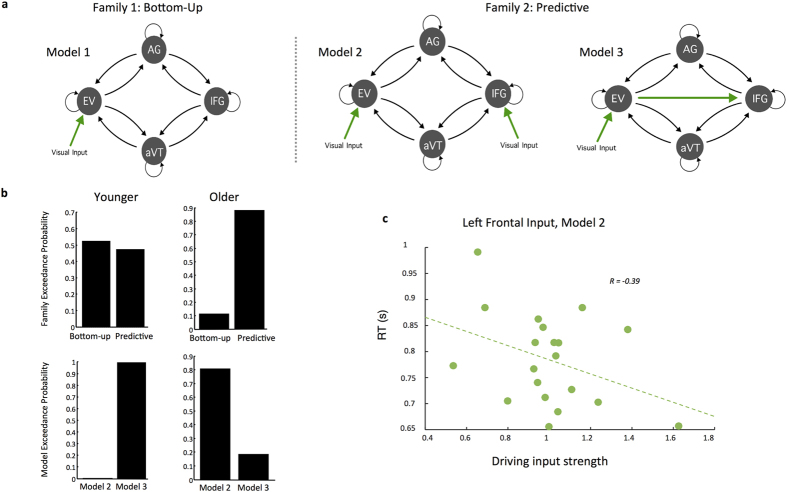
Model Comparison: Younger and Older Participants. (**a**) Time-series data were extracted bilaterally from four regions of interest: early visual cortex, anterior ventral temporal cortex, angular gyrus, and inferior frontal gyrus. Three models were constructed using the canonical microcircuit model in dynamic causal modeling. Model 1 included input to early visual cortex (EV), with reciprocal connections to the angular gyrus (AG) and anterior ventral temporal regions (aVT), which in turn shared reciprocal connections with the inferior frontal gyrus source (IFG). Model 2 included input to early visual cortex and inferior frontal gyrus. Model 3 included input to early visual cortex and a direct feedforward connection from early visual cortex to frontal cortex. These models were separated into 2 families defined by whether the model included traditional, bottom-up processing (model 1) or a fast, predictive signal to frontal cortex (models 2 and 3). (**b**) Family-level random-effects Bayesian model selection was used to compare model evidence for younger and older participants separately. Younger participants favored a bottom-up, hierarchical model with inputs to early visual cortex (family 1 exceedance probability: 0.526), while older participants overwhelmingly favored a model that included a fast, predictive signal to frontal cortex (family 2 exceedance probability: 0.883). To determine whether top-down predictions project directly to frontal cortex from thalamus, Model 2 was compared with Model 3 for both younger and older participants. Random-effects Bayesian model selection overwhelmingly favored Model 2 to Model 3 in older participants (Model 2 exceedance probability: 0.811), but Model 3 in younger participants (Model 3 exceedance probability: 0.997). (**c**) Pairwise linear correlation identified a significant (r = −0.39, p < 0.05) negative correlation between the strength of the input volley to left inferior frontal gyrus and naming speed for older participants.

While activation patterns for novel and repeated pictures within this cortical network have been studied extensively, little research has explored the role of the frontal cortex as the principle source of feedback predictions accompanying aging. We thus constructed three plausible models to account for the patterns of activity recorded at these 8 sources in our paradigm (Fig. 2a). All models included feedforward connections from early visual cortex to both anterior ventral temporal cortex and the angular gyrus. Both the anterior ventral temporal cortex and angular gyrus then provided feedforward connections to the inferior frontal gyrus. Backward connections ensured recurrent extrinsic connections between these same regions. The models differed in terms of the driving input which represents subcortical volleys timed at stimulus onset and initiates signal propagation throughout the network^18^. The models also differed as to whether frontal cortex provided fast feedback projections to signals propagating feedforward along the cortical hierarchy. Therefore, the models were allocated to two separate families (Fig. 2a), and family-level inference was used to test for this characteristic of interest between our two participant groups^20^. In family 1, input to the model entered early visual cortex, allowing signals to propagate forward to the angular gyrus and ventral temporal cortex, continuing on to frontal cortex (Fig. 2a). This model can be thought of as a traditional, bottom-up processing hierarchy. In family 2, two alternative models, models 2 and 3, accounted for predictive signaling from frontal cortex (Fig. 2a). In model 2, inputs entered both early visual cortex and frontal cortex directly. In model 3, inputs entered early visual cortex, and a direct feedforward connection from early visual cortex to frontal cortex allowed for signals to reach frontal cortex to provide top-down prediction signaling (Fig. 2a). We fit the DCMs to stimulus-evoked data over 1–450 msec peristimulus time. Using family-level random-effects Bayesian model selection (BMS), we compared which family had the best explanatory power within the younger and older cohorts separately. We found that family 1, with driving input to early visual cortex and a traditional bottom-up signaling hierarchy, showed the greatest family-level evidence for the younger participants (family 1 exceedance probability: 0.526), while family 2, which included fast predictive codes within frontal cortex, showed the greatest model evidence for older participants (family 2 exceedance probability: 0.883) (Fig. 2b).

**Figure 3:**
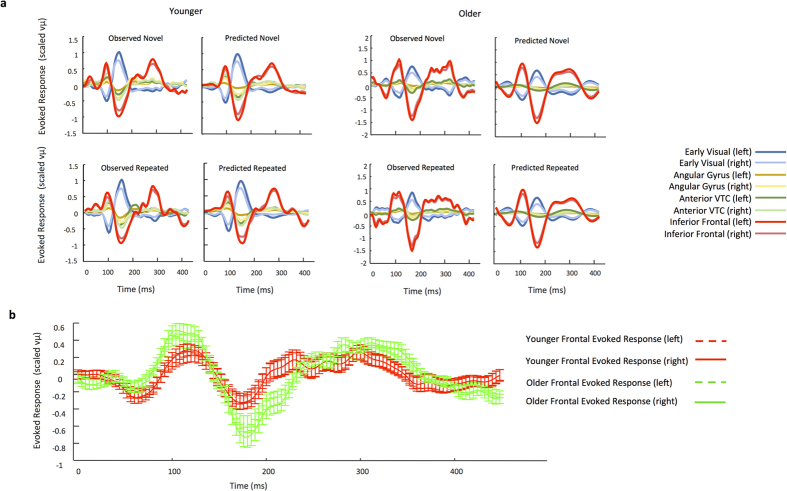
Model Fits and Frontal Evoked Activity. (**a**) Example DCM model fits for the 8 extracted time series for a single younger (left; fit = 0.98) and older (right; fit = 0.95) participant. For each, the left panel represents the evoked activity for each extracted time series, while the right panel is the optimized DCM-based fit. Responses to novel pictures are shown in the top panel and repeated pictures are shown in the bottom panel. The model effectively captured the dynamic properties of each extracted time series for both trial types in both the young and older cohorts. (**b**) Evoked activity in inferior frontal gyrus demonstrated a significantly larger amplitude and earlier response for older (green lines) compared with younger participants (red lines) for both novel and repeated images. Illustrated is the group mean and s.e.m.

To further explore the origin of this prefrontal driving input in our older cohort we examined which of the two predictive models that included early frontal cortex activity (models 2 and 3) provided the greatest explanatory power in our older cohort. Random-effects BMS was used to compare models 2 and 3 directly for our older participants. Model evidence strongly favored model 2 (model 2 exceedance probability: 0.811) (Fig. 3b), suggesting a direct subcortical route of visual inputs, which may result from projections from the mediodorsal nucleus of the thalamus^7^,^11^. A similar analysis was computed for younger participants, since the family level inference showed positive though not strong evidence in favor of family 1. This comparison of model 2 and 3 for our younger participants identified model 3 as providing the greatest explanatory power (model 3 exceedance probability: 0.997), consistent with a visual cortical initiation of network dynamics. In order to further validate the functional role of the direct frontal pathway in our older cohort, we sought to establish a behavioral correlation. We thus determined whether the strength of the modeled subcortical input volley to frontal cortex influenced reaction times in older participants. We found a significant negative correlation between input strength and reaction time, with older participants having a stronger input volley exhibiting faster naming speed (Fig. 3c). In both groups, our models accurately recapitulated the recorded brain dynamics observed in individuals (Fig. 3a). Also, these model evidences were consistent with observable temporal dynamics of evoked activity in frontal cortex, which showed an earlier and higher amplitude response for older compared with younger participants (Fig. 3b).

**Figure 4:**
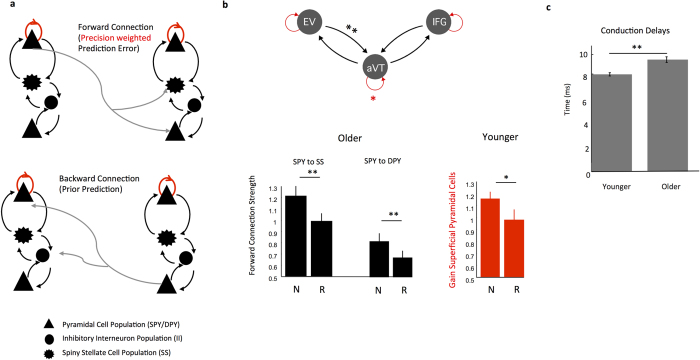
Predictive Codes in Ventral Visual Sources. (**a**) The canonical microcircuit model in DCM includes four distinct cell layers: superficial pyramidal cells, spiny stellates, inhibitory interneurons, and deep pyramidal cells. Superficial pyramidal cells carry feedforward prediction errors to both spiny stellate cells and deep pyramidal cells. Deep pyramidal cells carry feedback predictions to both superficial pyramidal cells and inhibitory interneurons. Gain is carried via the self connection (denoted here with a red, arrowed line) on superficial pyramidal cells. (**b**) We examined predictive codes (hypothesized to be greater for repeated compared to novel stimuli) for all ventral stream connections from early visual cortex to inferior frontal gyrus by harvesting the parameters encoding trial differences from the winning optimized DCMs. One-sample t-tests were computed to determine task modulations that were significantly different from zero, corrected for multiple comparisons. For older participants, predictive codes were carried in the strength of forward connections (weaker connections for repeated items) between early visual and anterior ventral temporal cortex (SPY to SS Novel Mean = 1.21 +/− 0.39 SD, SPY to SS Repeated Mean = 0.99 +/− 0.32; SPY to DPY Novel Mean = 0.81 +/− 0.33, SPY to DPY Repeated Mean = 0.67 +/− 0.28). For younger participants, predictive codes were carried in the gain of the self-connection on anterior ventral temporal cortex (Gain Novel = 1.16 +/− 0.27 SD, Gain Repeated = 0.98 +/− 0.43). (**c**) Behaviorally, aging was accompanied by a slowing in naming speed overall. We examined whether this slowing was reflected in conduction speed for forward, backward, or self-connections by harvesting the delay parameters from the winning optimized DCMs. All forward, backward, and self-connection conduction delays were averaged and then two-sample t-tests were computed between younger and older participants. We found significant (p < 0.01) slowing in forward connection conduction speed for older (Mean = 9.18 +/− 1.04 msec) compared with younger (Mean = 7.98 +/− 0.64 msec) participants.

Having identified a shift from the classical feedforward-initiated processing to top-down driven visual recognition in older adults, we then examined the nature of predictive codes encoded in extrinsic and intrinsic connections throughout the ventral stream for both younger and older participants. Predictive coding posits that top-down predictions, carried via deep pyramidal cells, serve to explain away prediction errors carried feedforward from earlier regions in the cortical hierarchy via superficial pyramidal cells. One elaborated predictive coding scheme proposed for cortical cell assemblies known as the Free Energy Principle^12^ additionally proposes that the size of forward prediction errors are modulated by their precision (the certainty of the prediction) and has been shown to map to the gain or ensemble synchronicity of superficial pyramidal cells^21^. The CMC model includes feedforward connections from superficial pyramidal cells to both spiny stellate cells and deep pyramidal cells, backward connections from deep pyramidal cells to both inhibitory interneurons and superficial pyramidal cells (Fig. 4a), as well as an intrinsic gain parameter which may control precision-weighting on error signals. Using this architecture, we tested whether repeated items induced extrinsic and intrinsic changes in neural connectivity commensurate with greater predictability. We particularly focused on the ventral visual stream connecting early visual regions to the inferior frontal gyrus, as the ventral stream has been shown to demonstrate repetition-suppression in traditional priming studies^13^,^14^,^15^ (Fig. 4b). Using the data-optimized parameter estimates for younger and older participants separately, we found evidence of reduced prediction error signaling for repeated compared with novel pictures in the ventral stream. Interestingly these effects comprised a reduction in forward connection strength from early visual cortex to the anterior ventral temporal cortex for older participants (Fig. 4b) while for younger participants, we found a reduction in the gain (i.e., decreased precision) of superficial pyramidal cells in anterior ventral temporal cortex for repeated compared to novel pictures (Fig. 4b). These findings are both commensurate with predictive coding under the Free Energy hypothesis where in older adults error propagation is reduced in early extrinsic pathways for predictable stimuli while for younger adults error propagation is reduced once the signal reaches the anterior temporal lobe.

Behaviorally, aging was accompanied by a slowing in naming speed for all items compared with younger participants. We were curious whether this overall slowing was evidenced by our model parameters and in particular whether it reflected a slowing of conduction velocity throughout the network generally. We tested the optimized conduction delay parameters^19^ in both feedforward and backward connections and found that within our modeled network a significant increase in conduction delays was present in the forward connections, for older compared to younger participants (Fig. 4c).

## Discussion

Aging is accompanied by profound changes in cognition with studies demonstrating changes in sensory perception^22^,^23^ learning^24^ and memory^25^ as well as reduced working and episodic memory performance^26^. While age-related changes in functional brain connectivity have been previously reported for the brain at rest^27^,^28^, our approach offers an assessment of the selective behavioral recruitment of active brain pathways. We were particularly interested in whether connections associated with predictive codes were differentially recruited by older adults, suggesting that improvements in internal generative models or stronger predictions about incoming sensory information accompany aging^24^. Our analysis was motivated by the confluence of findings of a posterior-anterior shift in brain activity with age^29^, as well as mounting functional evidence for predictive codes in the neural activity associated with visual perception^3^,^6^. A natural consequence of aging, which we assume amounts to a larger aggregate of visual inputs and repeated visual inputs, could result in hierarchical reorganization of perceptual network connectivity. In particular, we hypothesized that older individuals may exhibit increased recruitment of top-down signals. Our findings support this hypothesis and crucially point to a functional pathway that can readily facilitate this effect. We found that electrophysiological signals recorded from older participants were better accounted for by a cortical model of repeated visual object naming that included input to both early visual cortex and inferior frontal gyrus, while data from younger participants favored a model that included only early visual inputs.

While traditional models of object recognition suggest that object processing involves bottom-up signals that project feedforward along the ventral stream, more recent theories suggest that object recognition involves both bottom-up and top-down signaling^7^,^8^. According to one view, these bottom-up and top-down signals could be mediated via different subcortical white matter pathways or by direct early visual to prefrontal connections that then induce a top-down cascade^8^. Specifically, magnocellular projections, responding to low spatial frequency information available in the visual scene, transmit information to frontal cortex via one of these routes. These signals have been demonstrated to enhance processing speed and allow a first-pass analysis of incoming sensory information^7^. Supporting evidence has demonstrated frontal cortex activation immediately preceding activation in temporal cortex during repetition priming^7^, suggesting that frontal cortex predictions act upon incoming sensory information or prediction errors that are carried forward from early visual cortices. Our findings support a prefrontal input pathway for older participants that is independent of visual cortex and could arise instead from mediodorsal thalamus, where strong reciprocal connections with prefrontal regions are present^30^ and where a direct retinal input has recently been reported in primates^11^. In addition to our model comparison analysis, we found that the magnitude of the modeled subcortical volley to frontal cortex was inversely correlated with reaction time in our older cohort. That is, older participants responded faster when there was a greater input volley to inferior frontal gyrus. While in our current task, the data from the younger cohort showed greater statistical evidence for a bottom-up driven hierarchical network of responses, it is entirely possible that the frontal driving input pathway may be a feature of younger brains also. In future work we will test familiarity effects on this network connection which could reveal its emergence in younger brains, for example for tasks under greater predictive constraints.

According to the predictive coding framework, neural communication involves reciprocal message passing between hierarchically organized brain regions^4^,^31^. Critically, top-down predictions, thought to originate in deep pyramidal layers of neocortex, are generated to attempt to suppress prediction errors, while prediction errors can be tuned to have a greater or lesser bottom-up effect based on their precision^31^,^32^. Our findings highlight a change in the type of predictive code utilized by younger compared to older adults in the ventral stream. While older participants demonstrate an extrinsic effect of region-to-region connectivity commensurate with reduced errors, younger participants rely on a reduction in intrinsic precision or gain of cells within temporal lobe to stop error propagation during viewing of a repeated compared to novel object. This dichotomy is interesting given the proposed role of neuromodulators in effecting gain control^21^, and the decline in neuromodulatory activity with aging^33^. Thus, not only do we find a shift in terms of visual inputs in aging, we also observe a shift in how predictive codes may emerge. In particular, cortico-cortical mechanisms supplant a putative neuromodulatory mechanism. Finally, while our predictive coding hypothesis can account for altered connectivity patterns that promote facilitated priming effects, they do not account for the overall slowing of behavioral responses in our older individuals. Interestingly though, we find a slowing of conduction velocity along feedforward connections in these individuals. These model-based assays of the speed of cortico-cortical information propagation may reflect degraded fiber tract integrity, a prominent structural change in aging brains^34^. The present study focused on a comparison of early to late lifespan effects. In future work a characterization of the emergence of this pathway will require the study of middle-aged adults. This will help to elucidate whether the predictive framework adapts slowly with age or whether it is an effect that emerges in a step-like response in later years.

One limitation of the current study is that we relied on self-reported vision for all participants. Thus, we did not perform any visual acuity testing on participants, but relied on reported normal or corrected-to-normal vision for all participants. This could result in a greater decline in vision for older compared with younger participants, which might mean that peripheral degeneration accounts for or contributes to, the effects we report. For example, older participants may not have correctly identified the picture due to visual decline. However, in terms of the priming task, as long as participants were consistent in how they named the item, even if they could not identify it visually, then the name they used was irrelevant. Recent research has demonstrated that long-term object priming does not depend on explicit detection of object identity during the encoding stage^35^. Importantly, all participants demonstrated significant implicit memory via repetition priming: i.e., repeated items in this context are more predictable, leading to a reduced reaction time, compared with novel items.

## Methods

### Participants

Forty-four healthy participants gave written, informed consent and participated in the study. Participants were separated into two groups based on age (younger: N=23, mean age = 23.8 years, range = 18–36 years, 13 females; older: N = 21, mean age = 73.7 years, range = 66–91 years, 12 females). All participants were free from neurological or psychiatric disorders. All participants had self-reported normal or corrected to normal vision, though no data on participants’ last vision assessment by a professional clinician was collected. Protocols were approved by both the Virginia Polytechnic Institute and State University and Carilion Clinic ethics committees. All methods were carried out in accordance with approved guidelines.

### EEG Acquisition and Preprocessing

EEG recordings were collected using a DC amplifier (BrainAmp MR Plus, Brain Products GmbH Gilching Germany) and a 64-channel electrode system (actiCAP, Brain Products GmbH), referenced to the average of 64 channels. Impedances of all electrodes were confirmed to be <5 kΩ prior to data acquisition. During acquisition, data were sampled at 1000 Hz and online filtered at DC-250 Hz.

EEG data were collected during a picture-naming paradigm in which stimulus repetition was varied. A total of 200 full-color pictures of objects drawn from common categories (84 animals, 10 body parts, 74 foods, and 32 plants) were used in the experiment. Prior to scanning, participants were presented with an encoding phase during which 100 novel pictures of objects were shown. Participants were instructed to name each item covertly as quickly as possible, pressing a button to record reaction time. Each picture was presented for 2 s with a variable 1.5–2.5 s inter-stimulus interval during which a fixation cross was presented. Following an hour delay period during which the EEG system was set up, participants were scanned while they covertly named and button-pressed to 50 previously viewed pictures (repeated) and 50 new (novel) pictures. Covert naming was employed to minimize EEG artifacts, In line with previous studies employing picture-naming tasks^36^,^37^. Participants were seated approximately 40 inches (101.6 cm) from the viewing screen. Images were sized so that the longest dimension was 300 pixels and presented centrally on a 1024 × 768 pixel screen. On average, stimuli were 7 inches (17.8 cm) tall and 7.5 inches (19.1 cm) wide, subtending a visual angle of 5 degrees. Stimulus presentation time was identical to the encoding phase and presentation order was randomized across participants. In addition to covert naming, participants also performed an explicit recognition memory task in which they made old/new judgments on 50 previously viewed pictures (not overlapping with pictures presented during the covert naming task) and 50 novel pictures. Behavioral data from this task is presented here.

Offline, EEG data were first down-sampled to 250 Hz, then bandpass filtered from 2-58 Hz, epoched from -50 to 500 msec peristimulus time, then artifact corrected using a threshold of 2000 (uV) and a 1000 msec excision window. Identical preprocessing steps were used for both younger and older participants’ data. Following this initial preprocessing routine data were manually inspected and any remaining eyeblinks or artifacts were removed, then the data were low-pass filtered at 42 Hz. For data processing we used the analysis routines available in the academic freeware SPM12 (Wellcome Trust Centre for Neuroimaging, London, UK, http://www.fil.ion.ucl.ac.uk/spm/).

### Source Localization and Source Activity Extraction

The multiple sparse priors routine was used to identify wide-band (1–42 Hz) sources of activity from each participant’s sensor-level data over a post-stimulus event time window from 1–450 msec. Averaged ERPs for novel and repeated pictures were localized to 512 potential mesh points using a variational Bayesian approach following co-registration of sensor positions to a canonical template brain. The group inversion option was used to localize both novel and repeated images together, while constructing individual participant-level activation maps separately. No prior constraints on source location were used. Following the group inversion, statistical maps of group activity were computed separately for younger and older participants. Group-level 1-sample t-tests were computed for all pictures (i.e., novel and repeated combined) compared to baseline, thresholded at p < 0.05 FWE correction.

Group-level statistical activation maps demonstrated wide-band source activity in overlapping brain regions important for object naming in both younger and older participants (Fig. 1b). Our focus was on dorsal and ventral routes for object naming, motivated by previous repetition suppression effects during similar priming tasks^8^. We therefore proceeded with an 8-source (4 regions bilaterally) model of source activity for our DCM analysis using a ventral route from early visual cortex to inferior frontal gyrus passing through anterior ventral temporal cortex, as well as a dorsal route between visual and frontal cortices passing through superior temporal gyrus (see source locations below and in Fig. 1b). Source activity at each location of interest was extracted using SPM’s source extraction algorithm with a 5 mm radius, extracting single trials for both novel and repeated trials over the time window and frequency window specified in the initial group inversion. Subsequent analyses used these ‘virtual electrode’ electrophysiological signals.

### Dynamic Causal Modeling

We used the canonical microcircuit model (CMC) for DCM for electrophysiology to model brain connectivity^5^. The CMC includes excitatory and inhibitory extrinsic connection parameters from four distinct cell layers: superficial pyramidal cells, spiny stellate cells, deep pyramidal cells, and inhibitory interneurons (Fig. 4a). Within the model, superficial pyramidal cells encode and carry prediction errors to spiny stellate cells and deep pyramidal cells in a feedforward manner up the cortical hierarchy, while deep pyramidal cells carry top-down predictions in a feedback manner to both superficial pyramidal cells and inhibitory interneurons. This architecture was designed to test predictive coding within cortical hierarchies.

Thalamic (stimulus bound) input was modeled with a Gaussian bump function that drove activity in our models. The lowest level of our cortical hierarchy included bilateral early visual cortex (left: −34, −94, −6, right: 34, −92, 2). From early visual cortex, signals were passed via forward connections to both the angular gyrus (AG; left: −60 −31 20, right: 56 −36 18) and anterior ventral temporal cortex (aVTC; left: −46 −6 −32, right: 46, −8, −32). From both AG and aVTC, signals were passed via forward connections to inferior frontal gyrus (IFG; left: −50, 30, −2, right: 48, 36, 6). Backward connections ensured recurrent extrinsic connections, important for measuring predictions, with top-down inputs from IFG to both AG and aVTC, and both AG and aVTC to early visual cortex.

For the DCM analyses, EEG activity for the extracted time series were fitted over 1–450 msec peristimulus time in a wide frequency band from 1–42 Hz using an LFP model to capture event-related potentials of evoked activity. For computation efficiency, DCM optimizes a posterior density over free parameters (parameterized by its mean and covariance) via a standard variational Bayesian inversion procedure^38^. Three models were constructed to examine predictive coding in aging, varying the location of inputs to start the dynamics (Fig. 2a). The input strength scales a Gaussian bump function u(t), which activates the input cell layer in the neural mass model^19^, and is formally the driving input to a set of nonlinear differential equations. Mathematically this is given by C, such that


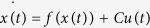


Where *x* are the neuronal states that generate the modeled ERP and *f* is the set of differential equations (see ref. 39 for a review of the dynamics). In the first model (Model 1), inputs were included to early visual cortex bilaterally. In the second model (Model 2), inputs were included to both early visual cortex and inferior frontal cortex bilaterally. In the third model (Model 3), inputs were included to early visual cortex bilaterally, with the addition of a direct feedforward connection from early visual cortex to frontal cortex. Trial specific modulatory effects (Novel – Repeated, so called ‘B’ parameters) were included on all extrinsic connections and on superficial pyramidal cell gain. For all models, initial DCMs were computed for each participant and model fits were assessed. Models with fits greater than 0.75 were then used to construct a group average (collapsing across age) of the estimated model parameters for each model separately. The means of the estimated parameters were then used to initialize a second set of DCMs for each participant with a model fit less than 0.75, initializing the starting location for each model separately. This ensured that the starting location for lower-fitting (i.e., <0.75) models were appropriate given the entire participant group and produced better fitting models to use in the comparison between participant groups. Family-level Bayesian model selection was used to compare two families of models directly. Family 1 included model 1, which can be considered to be a traditional, bottom-up account of signal propagation (Fig. 2a). Family 2 included both models 2 and 3, as these models included fast information passing to frontal cortex (Model 2 via direct input to frontal cortex, and Model 3 via a direct feedforward connection from early visual cortex to frontal cortex), allowing for top-down prediction signaling to interact with feedforward prediction error signals.

We harvested the parameter estimates from optimized DCMs for the winning model for each group separately to compare age-related effects at the model level. In particular, we were interested in the driving input strength within frontal cortex in older participants (i.e., DCM.Ep.C), task-related modulations within the ventral stream (i.e., DCM.Ep.B), the gain on self-connections within the ventral stream (i.e., DCM.Ep.G), and conduction delays in forward, backward, and self connections (DCM.Ep.D).

## Additional Information

**How to cite this article**: Gilbert, J. R. and Moran, R. J. Inputs to prefrontal cortex support visual recognition in the aging brain. *Sci. Rep.* **6**, 31943; doi: 10.1038/srep31943 (2016).
